# Neurological outcomes in children dead on hospital arrival

**DOI:** 10.1186/s13054-015-1132-1

**Published:** 2015-11-18

**Authors:** Yoshikazu Goto, Akira Funada, Yumiko Nakatsu-Goto

**Affiliations:** Department of Emergency and Critical Care Medicine, Kanazawa University Hospital, 13-1 Takaramachi, Kanazawa, 920-8641 Japan; Department of Cardiology, Yawata Medical Center, 12-7 I Yawata, Komatsu, 923-8551 Japan

## Abstract

**Introduction:**

Obtaining favorable neurological outcomes is extremely difficult in children transported to a hospital without a prehospital return of spontaneous circulation (ROSC) after out-of-hospital cardiac arrest (OHCA). However, the crucial prehospital factors affecting outcomes in this cohort remain unclear. We aimed to determine the prehospital factors for survival with favorable neurological outcomes (Cerebral Performance Category 1 or 2 (CPC 1–2)) in children without a prehospital ROSC after OHCA.

**Methods:**

Of 9093 OHCA children, 7332 children (age <18 years) without a prehospital ROSC after attempting resuscitation were eligible for enrollment. Data were obtained from a prospectively recorded Japanese national Utstein-style database from 2008 to 2012. The primary endpoint was 1-month CPC 1–2 after OHCA.

**Results:**

The 1-month survival and 1-month CPC 1–2 rates were 6.92 % (n = 508) and 0.99 % (n = 73), respectively. The proportions of the following prehospital variables were significantly higher in the 1-month CPC 1–2 cohort than in the 1-month CPC 3–5 cohort: age (median, 3 years (interquartile range (IQR), 0–14) versus 1 year (IQR, 0–11), *p* <0.05), bystander-witnessed arrest (52/73 (71.2 %) versus 1830/7259 (25.2 %), *p* <0.001), initial ventricular fibrillation (VF)/pulseless ventricular tachycardia (VT) rhythm (28/73 (38.3 %) versus 241/7259 (3.3 %), *p* <0.001), presumed cardiac causes (42/73 (57.5 %) versus 2385/7259 (32.8 %), *p* <0.001), and actual shock delivery (25/73 (34.2 %) versus 314/7259 (4.3 %), *p* <0.0001). Multivariate logistic regression analysis indicated that 2 prehospital factors were associated with 1-month CPC 1–2: initial non-asystole rhythm (VF/pulseless VT: adjusted odds ratio ( aOR), 16.0; 95 % confidence interval (CI), 8.05–32.0; pulseless electrical activity (PEA): aOR, 5.19; 95 % CI, 2.77–9.82) and bystander-witnessed arrest (aOR, 3.22; 95 % CI, 1.84–5.79). The rate of 1-month CPC 1–2 in witnessed-arrest children with an initial VF/pulseless VT was significantly higher than that in those with other initial cardiac rhythms (15.6 % versus 2.3 % for PEA and 1.2 % for asystole, *p* for trend <0.001).

**Conclusions:**

The crucial prehospital factors for 1-month survival with favorable neurological outcomes after OHCA were initial non-asystole rhythm and bystander-witnessed arrest in children transported to hospitals without a prehospital ROSC.

## Introduction

Out-of-hospital cardiac arrest (OHCA) is a leading cause of death and is an increasing public health concern in developed countries [1–3]. In recent population-based studies of pediatric OHCA the rates of survival with favorable neurological outcomes ranged from 4.3 % to 9.0 % [4–6], which are considerably lower than those for pediatric in-hospital cardiac arrest [7, 8]. Outcomes in children after cardiac arrest depend on a multitude of variables including age, comorbidities, initial cardiac rhythm, and other circumstances related to cardiac arrest, such as the time to return of spontaneous circulation (ROSC) [9, 10]. Goto et al. [10] previously demonstrated that three prehospital variables (prehospital ROSC, initial shockable rhythm, and witnessed arrest) were crucial predictors of 1-month outcomes in children who experienced OHCA. Of these variables, the most powerful predictor associated with favorable neurological outcomes was prehospital ROSC. However, the crucial prehospital factors for long-term survival with favorable neurological outcomes in OHCA children transported to hospitals without a prehospital ROSC are not clear.

This study aimed to determine the prehospital factors that influence the 1-month survival with favorable neurological outcomes in children transported to hospitals without a prehospital ROSC after OHCA.

## Methods

### Study design and data source

This investigation was a nationwide population-based observational study of all children (age <18 years) for whom resuscitation had been attempted after an OHCA in Japan between 1 January 2008 and 31 December 2012. Cardiac arrest was defined as the cessation of cardiac mechanical activities, as confirmed by the absence of signs of circulation [1]. The cause of arrest was presumed to be of cardiac origin, unless evidence suggested external causes (trauma, hanging, drowning, drug overdose, or asphyxia), respiratory diseases, cerebrovascular diseases, malignant tumors, or any other non-cardiac cause. The attribution of cause as non-cardiac or cardiac was made by the physicians in charge in collaboration with the emergency medical services (EMS) personnel. We considered all children who received resuscitation for analysis, regardless of whether or not the causes of cardiac arrest were traumatic. This study was approved by the Ethical Committee of Kanazawa University. According to the informed consent guideline in Japan [11], it is unnecessary to obtain informed consent from each patient to use secondary data from an anonymous database; therefore, this requirement for written informed consent was waived.

### EMS system in Japan

Japan has approximately 127 million residents in an area of 378,000 km^2^, approximately two thirds of which is uninhabited mountainous terrain. Details about the Japanese EMS system have been described previously [1, 12, 13]. Briefly, municipal governments provide EMS through approximately 800 fire stations with dispatch centers. The Fire and Disaster Management Agency (FDMA) of Japan supervises the nationwide EMS system, whereas each local EMS system is operated by the local fire station. Generally, an ambulance crew includes three EMS staff members, including at least one emergency life-saving technician (ELST) [13]. ELSTs are allowed to use several resuscitation methods, including use of semi-automated external defibrillators, insertion of a supraglottic airway device (laryngeal mask airway, laryngeal tube, and esophageal-tracheal twin-lumen airway device), insertion of a peripheral intravenous line, and administration of Ringer lactate solution. Since July 2004, only specially trained ELSTs are permitted to insert a tracheal tube, and since April 2006, they have been permitted to administer intravenous epinephrine in the field under the instruction of an online physician. All EMS providers perform cardiopulmonary resuscitation (CPR) according to the Japanese CPR guidelines [14, 15]. As EMS personnel in Japan are legally prohibited from terminating resuscitation in the field, most OHCA patients receive CPR from EMS providers and are transported to hospitals, except in specific situations (that is, decapitation, incineration, decomposition, rigor mortis or dependent cyanosis) [16]. The duration of on-scene effort by EMS personnel before transport is initiated is not predetermined.

### Data collection and quality control

The FDMA launched a prospective, population-based observational study that includes all OHCA patients who have received EMS in Japan since January 2005 [1, 10, 13]. EMS personnel at each center record data for OHCA patients, with the cooperation of the physician in charge, using an Utstein-style template [17]. The data are transferred to their fire stations and are then integrated into the registry system on the FDMA database server. The data are checked for consistency by the computer system and are confirmed by FDMA personnel. If the data form is incomplete, the FDMA returns it to the respective fire station, where the form is completed [1]. All data are stored in the nationwide database developed by the FDMA for public use. The FDMA granted permission to analyze this database and provided all the anonymous data to our research group. The main variables included in the dataset are sex, age, cause of arrest (presumed cardiac etiology or not), bystander witness status, bystander CPR, use of automated external defibrillator, initial identified cardiac rhythm, bystander category (that is, the presence or absence of a bystander, or whether the bystander was a layperson or an EMS staff member), achievement of ROSC before arrival at the hospital, time of the emergency call, time of vehicle arrival at the scene, time of ROSC, time of vehicle arrival at the hospital, survival and neurological outcome at 1 month after cardiac arrest. The neurological outcome was defined according to the cerebral performance category (CPC) scale: category 1, good cerebral performance; category 2, moderate cerebral disability; category 3, severe cerebral disability; category 4, coma or vegetative state; and category 5, death [17]. The CPC categorization was determined by the physician in charge. The call-to-response time was calculated as the time from the emergency call to the time of vehicle arrival at the scene. The call-to-hospital-arrival time was calculated as the time from the emergency call to the time of vehicle arrival at the hospital.

### End points

The primary study end point was 1-month survival with favorable neurological outcome (defined as a CPC of 1 or 2) [17]. The secondary end point was survival at 1 month after OHCA.

### Statistical analysis

Kolmogorov–Smirnov–Lilliefors tests were performed to evaluate the distributions of continuous variables, and we found that all continuous variables were not normally distributed (all *p* <0.01). Therefore, the Kruskal–Wallis tests for continuous variables and the chi square (χ^2^) test for categorical variables were performed to compare the characteristics or outcomes between the cohorts. The Cochran-Armitage trend test was applied to compare the outcomes 1 month after cardiac arrest according to initial cardiac rhythm in both bystander-witnessed and bystander-unwitnessed arrests. Multivariate logistic regression analyses including nine variables were performed to assess the factors contributing to 1-month survival and 1-month CPC 1–2 for all eligible patients. The nine selected variables included year, age, sex (boys or girls), bystander-witnessed arrest (yes or no), bystander CPR (yes or no), presumed cardiac cause (yes or no), initial cardiac rhythm (ventricular fibrillation (VF), pulseless ventricular tachycardia (VT), pulseless electrical activity (PEA), or asystole), call-to-response time, and call-to-hospital-arrival time for the model as independent variables. These models yielded concordance statistics of 0.71 for 1-month survival and 0.87 for 1-month CPC 1–2, which indicated good discrimination. In these multivariate logistic regression analyses for outcomes, we classified the following three continuous variables into three or four categories: age (<1, 1–11, and 12–18 years), call-to-response time (≤5, 6–10, and ≥11 minutes), and call-to-hospital-arrival time (≤20, 21–30, 31–40, and ≥41 minutes).

Continuous variables are expressed as means and standard deviations. Categorical variables are expressed as percentages. As an estimate of effect size and variability, we report odds ratios (ORs) with 95 % confidence intervals (CIs). All statistical analyses were performed using the JMP statistical package version Pro 11 (SAS Institute Inc., Cary, NC, USA). All tests were two-tailed, and a *p* value <0.05 was considered statistically significant.

## Results

**Fig. 1:**
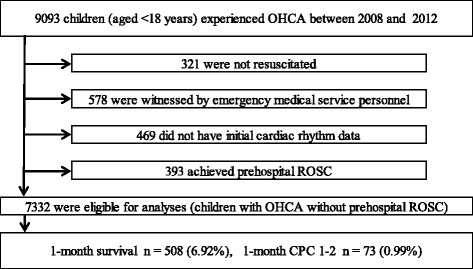
Flow diagram of selection of participants. *CPC* cerebral performance category, *OHCA* out-of-hospital cardiac arrest, *ROSC* return of spontaneous circulation

During the 5-year study period, 9,093 children were documented in the database. We excluded patients who were witnessed by EMS personnel and had any prehospital ROSC, no matter how transient, and finally considered 7,332 (80.6 %) children eligible for enrollment. Figure 1 shows a flow diagram depicting the inclusion and exclusion criteria for subjects in the present study. The overall 1-month survival and survival with favorable neurological outcome (CPC 1–2) rates were 6.92 % (508/7,332) and 0.99 % (73/7,332), respectively.

Table 1 shows the baseline characteristics of study patients according to 1-month survival after OHCA. The proportions of bystander-witnessed arrest, bystander CPR, initial VF/pulseless VT, and prehospital actual shock delivery in the survival cohort were significantly higher than those in the death cohort. Call-to-response and call-to-hospital-arrival times in the survival cohort were significantly lower than those in the death cohort.

**Table 1:** Baseline characteristics of the study patients according to one-month survival

Characteristic	Survival		Death		*P* value
	(n = 508)		(n = 6,824)		
Year					
2008	124	(24.4)	1,681	(24.6)	
2009	83	(16.3)	1,283	(18.8)	
2010	92	(18.1)	1,317	(19.3)	0.31
2011	99	(19.5)	1,291	(18.9)	
2012	110	(21.7)	1,252	(18.4)	
Age, years, median (IQR 1–3)	1.0	(0–9)	1.0	(0–11)	0.65
<1	200	(39.4)	2,963	(43.4)	
1–11	206	(40.5)	2,187	(32.1)	<0.001
12–18	102	(20.1)	1,674	(24.5)	
Boys	287	(56.5)	4,170	(61.1)	0.04
Bystander-witnessed arrest	212	(41.7)	1,670	(24.5)	<0.001
Bystander CPR	313	(61.6)	3,690	(54.1)	<0.01
Presumed cardiac cause	149	(29.3)	2,278	(33.4)	0.06
Initial cardiac rhythm					
Ventricular fibrillation/pulseless ventricular tachycardia	57	(11.2)	212	(3.1)	<0.001
Pulseless electrical activity	147	(28.9)	844	(12.4)	
Asystole	304	(59.8)	5,768	(84.5)	
Prehospital actual shock delivery	53	(10.4)	286	(4.2)	<0.001
Call-to-response time, minutes, median (IQR 1–3), n = 7,321 (99.8 %)	6.0	(5–8)	7.0	(5–9)	<0.01
≤5	168	(33.1)	1,977	(28.9)	0.01
6–10	290	(57.1)	3,915	(57.4)	
≥11	48	(9.5)	923	(13.5)	
Call-to-hospital-arrival time, minutes, median (IQR 1–3), n = 7,291 (99.4 %)	24.0	(19–31)	27.0	(21–35)	<0.001
≤20	154	(30.3)	1,460	(21.4)	<0.001
21–30	218	(42.9)	2,852	(41.8)	
31–40	89	(17.5)	1,526	(22.4)	
≥41	40	(7.9)	952	(14.0)	

Values are reported as number (%) unless indicated otherwise. *CPR* cardiopulmonary resuscitation, *IQR* interquartile range

Table 2 shows the baseline characteristics of the study patients according to 1-month neurological outcomes after OHCA. In the CPC 1–2 cohort, patients were 2 years older and had higher proportions of bystander-witnessed arrest, bystander CPR, presumed cardiac cause, initial VF/pulseless VT, and prehospital actual shock delivery than those in the CPC 3–5 cohort. Call-to-response and call-to-hospital-arrival times in the CPC 1–2 cohort was shorter that those in the CPC 3–5 cohort.

**Table 2:** Baseline characteristics of the study patients according to one-month neurological outcomes

Characteristic	CPC 1–2		CPC 3–5		*P* value
	(n = 73)		(n = 7,259)		
Year					
2008	17	(23.3)	1,788	(24.6)	
2009	12	(16.4)	1,354	(18.7)	
2010	13	(17.8)	1,396	(19.2)	0.93
2011	15	(20.6)	1,375	(18.9)	
2012	16	(21.9)	1,346	(18.5)	
Age, years, median (IQR 1–3)	3.0	(0–14)	1.0	(0–11)	0.019
<1	22	(30.1)	3,141	(43.3)	
1–11	22	(30.1)	2,371	(32.7)	<0.01
12–18	29	(39.7)	1,747	(24.1)	
Boy	42	(57.5)	4,415	(60.8)	0.56
Bystander-witnessed arrest	52	(71.2)	1,830	(25.2)	<0.001
Bystander CPR	48	(65.8)	3,955	(54.5)	0.054
Presumed cardiac cause	42	(57.5)	2,385	(32.9)	<0.001
Initial cardiac rhythm					
Ventricular fibrillation/pulseless ventricular tachycardia	28	(38.4)	241	(3.3)	<0.001
Pulseless electrical activity	25	(34.3)	966	(13.3)	
Asystole	20	(27.4)	6,052	(83.4)	
Prehospital actual shock delivery	25	(34.3)	314	(4.3)	<0.001
Call-to-response time, minutes, median (IQR 1–3), n = 7,321 (99.8 %)	6.0	(5–8)	7.0	(5–9)	0.06
≤5	24	(32.9)	2,121	(29.2)	0.41
6–10	43	(58.9)	4,162	(57.3)	
≥11	6	(8.2)	965	(13.3)	
Call-to-hospital-arrival time, minutes, median (IQR 1–3), n = 7,291 (99.4 %)	24.0	(19–30)	27.0	(21–34)	<0.01
≤20	20	(27.4)	1,594	(22.0)	0.08
21–30	36	(49.3)	3,034	(41.8)	
31–40	12	(16.4)	1,603	(22.1)	
≥41	4	(5.5)	988	(13.6)	

Values are reported as number (%) unless indicated otherwise. *CPC* cerebral performance category, *CPR* cardiopulmonary resuscitation, *IQR* interquartile range

Table 3 shows the results of multivariate logistic regression analyses including nine variables to determine the factors associated with 1-month survival and 1-month CPC 1–2. Five of the nine variables were associated with increased odds of survival: age (<12 years old), bystander-witnessed arrest, bystander CPR, initial non-asystole cardiac rhythm, and call-to-hospital-arrival time (<31 minutes). The highest adjusted OR was initial VF/pulseless VT with an adjusted OR of 5.16 (95 % CI, 3.63–7.27) compared with initial asystole rhythm. Two of the nine variables were associated with increased odds of CPC 1–2: bystander-witnessed arrest and initial non-asystole cardiac rhythm. The highest adjusted OR was for initial VF/pulseless VT with an adjusted OR of 16.0 (95 % CI, 8.06–32.0) followed by initial PEA (adjusted OR, 5.19; 95 % CI, 2.77–9.82) and bystander-witnessed arrest (adjusted OR, 3.22; 95 % CI, 1.84–5.79).

**Table 3:** Results of multivariate logistic regression analyses for variables associated with one-month outcomes

Variables	Adjusted OR (95 % CI)			
	1-month survival		1-month CPC 1-2	
Year				
2008	Reference		Reference	
2009	0.87	(0.64–1.17)	1.09	(0.49–2.38)
2010	0.93	(0.69–1.24)	1.18	(0.53–2.54)
2011	1.06	(0.79–1.40)	1.26	(0.59–2.64)
2012	1.25	(0.94–1.65)	1.50	(0.72–3.12)
Age, years				
<1	1.35	(1.04–1.77)	0.69	(0.37–1.29)
1–11	1.61	(1.25–2.09)	0.68	(0.37–1.23)
12–18	Reference		Reference	
Boy	0.84	(0.69–1.01)	0.78	(0.48–1.29)
Bystander-witnessed arrest	1.62	(1.32–2.00)	3.22	(1.84–5.79)
Bystander CPR	1.41	(1.16–1.71)	1.49	(0.90–2.53)
Presumed cardiac cause	0.68	(0.54–0.84)	1.69	(0.99–2.87)
Initial cardiac rhythm				
Ventricular fibrillation/pulseless ventricular tachycardia	5.16	(3.63–7.27)	16.0	(8.06–32.0)
Pulseless electrical activity	2.83	(2.26–3.54)	5.19	(2.77–9.82)
Asystole	Reference		Reference	
Call-to-response time, minutes				
≤5	1.35	(0.95–1.95)	1.25	(0.50–3.58)
6–10	1.25	(0.90–1.77)	1.19	(0.52–3.26)
≥11	Reference		Reference	
Call-to-hospital-arrival time, minutes				
≤20	2.21	(1.53–3.27)	2.81	(0.99–10.1)
21– 30	1.61	(1.13–2.33)	2.31	(0.87–7.94)
31– 40	1.24	(0.84–1.85)	1.55	(0.52–5.66)
≥41	Reference		Reference	

*CI* confidence interval, *CPC* cerebral performance category, *CPR* cardiopulmonary resuscitation, *OR* odds ratio

**Fig. 2:**
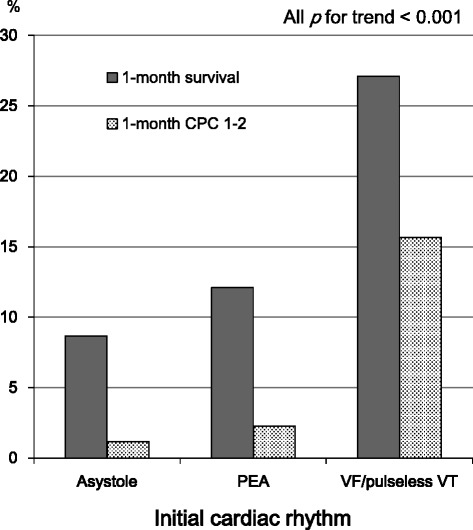
One-month outcomes according to the initial rhythm in bystander-witnessed out-of-hospital cardiac arrest. *CPC* cerebral performance category, *PEA* pulseless electrical activity, *VF* ventricular fibrillation, *VT* ventricular tachycardia

Figure 2 shows the rates of 1-month outcomes according to the initial cardiac rhythm in bystander-witnessed OHCA. Overall rates of 1-month survival and 1-month CPC 1–2 in bystander-witnessed OHCA were 11.3 % (212/1,882) and 2.76 % (52/1,882), respectively. The proportions of initial cardiac rhythm were 63.1 % (n = 1187) in asystole, 28.1 % (n = 529) in PEA, and 8.8 % (n = 166) in VF/pulseless VT, respectively. The rates of 1-month survival and 1-month CPC 1–2 were 8.7 % (103/1,187) and 1.2 % (14/1,187) in initial asystole, 12.1 % (64/529) and 2.3 % (12/529) in initial PEA, and 27.1 % (45/166) and 15.6 % (26/166) in initial VF/pulseless VT, respectively (*p* for trend <0.001 for all).

**Fig. 3:**
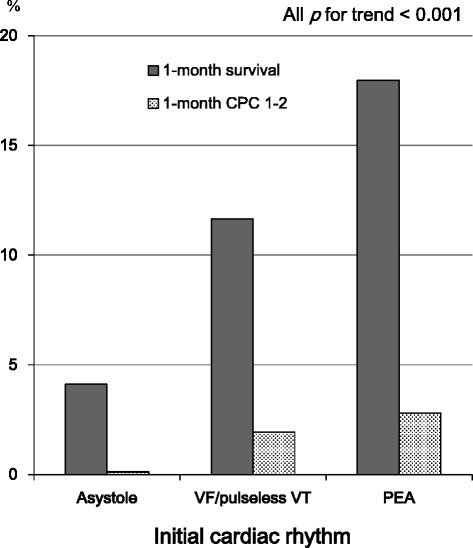
One-month outcomes according to the initial rhythm in bystander-unwitnessed out-of-hospital cardiac arrest. *CPC* cerebral performance category, *PEA* pulseless electrical activity, *VF* ventricular fibrillation, *VT* ventricular tachycardia

Figure 3 shows the rates of 1-month outcomes according to the initial cardiac rhythm in bystander-unwitnessed OHCA. The overall rates of 1-month survival and 1-month CPC 1–2 in bystander-unwitnessed OHCA were 5.43 % (296/5,450) and 0.39 % (21/5,450), respectively. The proportions of initial cardiac rhythm were 89.6 % (n = 4,885) in asystole, 1.9 % (n = 103) in VF/pulseless VT, and 8.5 % (n = 462) in PEA, respectively. The proportion of initial VF/pulseless VT rhythm in bystander-unwitnessed arrest was lower than that in bystander-witnessed arrest (1.9 % (103/5,450) versus 8.8 % (166/1,882), *p* <0.001). The proportion of initial asystole in bystander-unwitnessed arrest was significantly higher than that in bystander-witnessed arrest (89.6 % versus 63.1 %, *p* <0.001). The rates of 1-month survival and 1-month CPC 1–2 were 4.11 % (201/4,885) and 0.12 % (6/4,885) in initial asystole, 11.7 % (12/103) and 1.94 % (2/103) in initial VF/pulseless VT, and 18.0 % (83/462) and 2.81 % (13/462) in initial PEA, respectively (*p* for trend <0.001 for all).

## Discussion

In children transported to hospitals without a prehospital ROSC after OHCA, the present analysis demonstrates that the crucial prehospital variables for 1-month survival with favorable neurological outcomes after OHCA were initial non-asystole rhythm and bystander-witnessed arrest. When these two crucial predictors were present, approximately 1 of every 6.5 patients with initial VF/pulseless VT rhythm had 1-month survival with favorable neurological outcomes after OHCA without a prehospital ROSC.

In adults with OHCA, Sasson et al. [3] indicated that ROSC in the field is the most powerful predictor associated with survival from OHCA followed by EMS-witnessed arrest, initial VF/pulseless VT, bystander CPR, bystander-witnessed arrest, and initial non-asystole rhythm. Moreover, in their paper, the absence of prehospital ROSC indicates that patients with OHCA will not likely survive with favorable neurological outcomes. In children with OHCA, no predictors of out-of-hospital resuscitation success have been established [18, 19]. Excluding a prehospital ROSC, several variables have been suggested as a predictor for outcomes after OHCA in children: age [20, 21], bystander CPR [20, 22, 23], bystander-witnessed arrest [10, 20–23], initial VF [11, 24–26,], and earlier initiation of CPR by EMS personnel [21]. In our study, 1-month survival and 1-month CPC 1–2 rates of children with OHCA without a prehospital ROSC were very poor: 6.92 % and 0.99 %, respectively (Fig. 1). These results were consistent with those of Sasson’s report of OHCA adults without a prehospital ROSC [3]. However, even in such difficult conditions, the presence of the abovementioned two crucial factors (initial non-asystole rhythm and bystander-witnessed arrest) would increase the rates of survival with favorable neurological outcomes. In bystander-witnessed OHCAs in our present study, the rate of 1-month CPC 1–2 for children with initial VF/pulseless VT was approximately 13-fold higher than that for children with initial asystole (15.7 % versus 1.18 %, Fig. 2). However, the proportion of initial VF/pulseless VT in our study subjects was very low (3.67 %, 269/7,332). In bystander-unwitnessed OHCAs in our present study, the rate of 1-month CPC 1–2 for children with initial PEA was approximately 23-fold higher than that for children with initial asystole (2.81 % versus 0.12 %, Fig. 3). Interestingly, children who experienced bystander-unwitnessed arrest with initial VF/pulseless VT had poor 1-month outcomes compared to those with initial PEA (Fig. 3). This may be a direct consequence of the majority of arrests being of non-cardiac origin (66.9 %, 4,905/7,332) and being unwitnessed, leading to progressive hypoxia due to respiratory associated diseases and ultimately to cardiac arrest with arrhythmia.

The proportion of initial VF/pulseless VT was reported to range from 2.0 % to 36.0 % in previous studies of pediatric OHCA [4, 5, 10, 20, 21, 24]. Some possible reasons for this difference in the proportions among studies may be due to various EMS systems, definitions of OHCA, and inclusion criteria in pediatric OHCA, such as witnessed arrest or not, age of subjects, etiology of arrest, and so on. Previous studies from Japan reported that the proportion of initial VF/pulseless VT rhythm was ranging from 3.9 % to 4.9 % in all children with OHCA [10, 21, 25], and was 14.7 % [6] in children with bystander-witnessed OHCA. The presence of initial VF/pulseless VT rhythm is correlated with a witnessed arrest, bystander CPR, use of an automated external defibrillator, and call-to-response time and was associated with the presence of sustained ROSC in OHCA children [5, 26]. Therefore, it is reasonable that the proportion of initial VF/pulseless VT rhythm in our present study (3.67 %) for children without a prehospital ROSC was lower than that in previous studies in Japan. Moreover, in our present study, the proportion of initial VF/pulseless VT rhythm in bystander-unwitnessed arrest was lower than that in bystander-witnessed arrest (1.9 % versus 8.8 %, *p* <0.001). This result is consistent with a previous report that a shockable rhythm depends on the time interval since the onset of arrest [27].

Goto et al. [28] demonstrated that in adults transported to a hospital without a prehospital ROSC after OHCA, nine crucial factors were associated with increased ORs of 1-month favorable neurological outcomes: (1) initial non-asystole rhythm, (2) age <65 years, (3) EMS-witnessed arrest, (4) bystander-witnessed arrest, (5) physician-staffed ambulance, (6) call-to-response time <5 minutes, (7) prehospital shock delivery, (8) call-to-hospital-arrival time <24 minutes, and (9) presumed cardiac cause. In the present study aimed at determining the crucial factors for favorable neurological outcomes in pediatric OHCA without a prehospital ROSC, we found that only two prehospital factors (initial non-asystole rhythm and bystander-witnessed arrest) were associated with 1-month outcomes. Various factors may contribute to this difference between adults and children with cardiac arrest: etiology and pathophysiology of cardiac arrests, age-specific incidence differences, quality of bystander CPR, in-hospital care, preexisting conditions (underlying disease and degree of illness), and lifestyle and environmental factors [7, 25, 29].

In adults who experienced OHCA following futile resuscitation in the field, termination of resuscitation in the out-of-hospital setting has become more widespread [18]. In pediatric OHCA, however, termination of resuscitation by EMS personnel in the field is not generally accepted [18, 19]. EMS personnel in Japan are forbidden to implement termination of resuscitation for both adults and children with OHCA in the field [10, 14, 30]. In our present study, bystander-unwitnessed children with initial asystole who did not achieve prehospital ROSC had a 1-month survival rate of 4.11 % (Fig. 3). An objective criterion for medical futility was defined for interventions and drug therapy imparting a less than 1 % chance of survival [31], and this level remains the basis for current futility research [18]. Therefore, our results show that resuscitation for children, even in such difficult situations, were not futile. Thus, our study supports the 2010 guidelines for pediatric OHCA to not terminate resuscitation in the field, consider transportation to a hospital, and continue resuscitation [14, 18, 19]. The outcome for pediatric traumatic OHCA remains poor, although children with OHCA from other causes are more likely to survive than adults [10, 13, 23, 32]. A recently published manuscript [23] stated that if the resuscitation for traumatic pediatric OHCA has already exceeded 30 minutes, and the nearest facility is more than 30 minutes away, involvement of family of these children in the decision-making process with assistance and guidance from medical professionals should be considered as part of an emphasis on family-centered care, because of its inevitable poor outcome. Considering such circumstances, there is a need for more research and study to develop and implement termination-of-resuscitation protocols for children with OHCA.

### Study limitations

Our study has potential limitations. First, our database lacked detailed data to permit further risk adjustment for outcomes (e.g., comorbid disease, location of OHCA occurrence, quality of EMS personnel, CPR quality, regional variations among EMS centers, in-hospital medication, and availability of specialists in emergency care (cardiologists)). This is attributable to our study design of a retrospective record review. Second, although we used a uniform data collection procedure based on the Utstein-style guidelines for reporting cardiac arrest, a large sample size, and a population-based design, we cannot exclude the possibility of uncontrolled confounders. Third, as with all epidemiological studies, the integrity, validity, and ascertainment bias of the data are potential limitations. Particularly, the results of assessment of neurological status of the younger children using the CPC scale may have differed among the physicians in charge. Fourth, caution must be exercised when generalizing these results to other EMS systems because the present study was not a randomized controlled trial. Finally, there is a possibility that some patients may have had sudden infant death syndrome, which is a common etiology for arrest followed by trauma and respiratory disease [33], due to the lack of precise data on the causes of cardiac arrest.

## Conclusions

The crucial prehospital factors for 1-month survival with favorable neurological outcomes after OHCA were initial non-asystole rhythm and bystander-witnessed arrest in children transported to a hospital without a prehospital ROSC.

## Key messages

- We determined the prehospital factors that influence 1-month survival with favorable neurological outcomes in children transported to hospitals without a prehospital ROSC after an OHCA, using a prospectively recorded nationwide Utstein-style Japanese database.
- The rates of 1-month survival and 1-month CPC 1–2 in OHCA children without a prehospital ROSC were extremely poor (6.92 % and 0.99 %, respectively).
- Two prehospital factors were independently associated with increased odds of 1-month survival with favorable neurological outcomes: initial non-asystole rhythm (VF/pulseless VT and PEA) and bystander-witnessed arrests.
- When two crucial key factors were present, the rates of 1-month CPC 1–2 were 15.6 % for initial VF/pulseless VT and 2.3 % for initial PEA, respectively.

## Abbreviations

CI: confidence interval
CPC: cerebral performance category
CPR: cardiopulmonary resuscitation
ELST: emergency life-saving technician
EMS: emergency medical services
FDMA: Fire and Disaster Management Agency
OHCA: out-of-hospital cardiac arrest
OR: odds ratio
PEA: pulseless electrical activity
ROSC: return of spontaneous circulation
VF: ventricular fibrillation
VT: ventricular tachycardia
